# Beta-Defensin 2 and 3 Promote Bacterial Clearance of *Pseudomonas aeruginosa* by Inhibiting Macrophage Autophagy through Downregulation of Early Growth Response Gene-1 and c-FOS

**DOI:** 10.3389/fimmu.2018.00211

**Published:** 2018-02-13

**Authors:** Yongjian Wu, Dandan Li, Yi Wang, Xi Liu, Yuanqing Zhang, Wenting Qu, Kang Chen, Ngiambudulu M. Francisco, Lianqiang Feng, Xi Huang, Minhao Wu

**Affiliations:** ^1^Program of Pathobiology and Immunology, Fifth Affiliated Hospital, Zhongshan School of Medicine, Sun Yat-sen University, Guangzhou, China; ^2^Department of Gastroenterology, Guangzhou Women and Children’s Medical Center, Guangzhou, China; ^3^Key Laboratory of Tropical Diseases Control, Sun Yat-sen University, Ministry of Education, Guangzhou, China; ^4^School of Pharmaceutical Sciences, Sun Yat-sen University, Guangzhou, China; ^5^Division of Clinical Laboratory, Zhongshan Hospital of Sun Yat-sen University, Zhongshan, China

**Keywords:** beta-defensins, *Pseudomonas aeruginosa*, macrophages, autophagy, bacterial eradication

## Abstract

Beta-defensins 2 and 3 (BD2 and BD3) are inducible peptides present at the sites of infection, and they are well characterized for their antimicrobial activities and immune-regulatory functions. However, no study has thoroughly investigated their immunomodulatory effects on macrophage-mediated immune responses against *Pseudomonas aeruginosa* (PA). Here, we use THP-1 and RAW264.7 cell lines and demonstrate that BD2 and BD3 suppressed macrophage autophagy but enhanced the engulfment of PA and Zymosan bioparticles as well as the formation of phagolysosomes, using immunofluorescence staining and confocal microscopy. Plate count assay showed that macrophage-mediated phagocytosis and intracellular killing of PA were promoted by BD2 and BD3. Furthermore, microarray and real-time PCR showed that the expression of two genes, early growth response gene-1 (EGR1) and c-FOS, was attenuated by BD2 and BD3. Western blot revealed that BD2 and BD3 inhibited the expression and nuclear translocation of EGR1 and c-FOS. Knockdown of EGR1 and c-FOS by siRNA transfection suppressed macrophage autophagy before and after PA infection; while overexpression of these two transcription factors enhanced autophagy but reversed the role of BD2 and BD3 on macrophage-mediated PA eradication. Together, these results demonstrate a novel immune defense activity of BD2 and BD3, which promotes clearance of PA by inhibiting macrophage autophagy through downregulation of EGR1 and c-FOS.

## Introduction

*Pseudomonas aeruginosa* (PA) is an extracellular Gram-negative bacterium commonly found in the environment (1). PA is a causative agent of various opportunistic infections, including keratitis in contact lens wearers (2) and nosocomial infections in cystic fibrosis patients (3) or immunodeficiency individuals (4). The pathogenesis of the above-mentioned diseases largely results from various bacterial virulence factors, such as exoenzyme ExoU, endotoxin lipopolysaccharide (LPS), and exotoxin, which have been implicated in host cell death and tissue damage (3, 5). In the past decade, studies have been focused on host immunity against PA, because of the increased challenge of drug resistance in traditional antibiotic therapies (6).

Beta-defensins (BDs) have been reported as an important component in the host innate immunity against microbial invasion (7, 8). BDs are small cationic antimicrobial peptides (AMPs) that are mainly produced by epithelial cells and immunocytes. BDs exert broad-spectrum antimicrobial activity against bacteria (Gram positive and Gram negative), fungi, and some enveloped viruses (2, 7, 8). It is also reported that BDs are capable of electrostatically interacting with negatively charged components in microbial cell walls, by increasing the permeability of cell walls and ultimately leading to a host cell death (9). In addition to their antimicrobial function, BDs also function as inflammatory mediators, with impact on the immune activation of epithelial cells and immunocytes (10, 11), production of inflammatory cytokines (12, 13), as well as induction of chemotaxis (7, 14). For instance, Beta-defensins 2 (BD2) has been shown to chemoattract immature dendritic cells or memory CD4^+^ T cells through C–C motif chemokine receptor 6 (7), while beta-defensins 3 (BD3) and BD4 recruit macrophages dependent on cysteine residues (14). Furthermore, BDs can modulate inflammatory responses, but their regulatory roles remain controversial. Some studies have shown that BD3 induced pro-inflammatory cytokines, such as interleukin 6 (IL-6), IL-8, and tumor necrosis factor (TNF) *via* toll-like receptors (TLRs)/MyD88-dependent signaling (11, 15); whereas another study revealed that BD3 suppressed IL-6 and TNF in both human and mouse macrophages upon stimulation with TLR4 ligand or LPS (16). Using an *in vivo* murine model of PA keratitis, we have previously demonstrated that BD2 and BD3 together reduced corneal inflammation as well as bacterial load (17, 18). Though the evidence implicating BDs in bacterial pathogenesis is mounting, little is known regarding the immunoregulatory mechanism of BD2 and BD3 in bacterial clearance.

As one of the major types of phagocytes in the innate immune system, macrophages have been shown to eradicate invading pathogens by engulfing (phagocytosis) and destroying the intracellular microbes (19). Besides the phagocytosis and killing of microbes, autophagy (macroautophagy) is another conserved cellular process associated with innate immune response (20). Indeed, the activation of autophagy in macrophages is often considered as a protective strategy against intracellular pathogens, such as *Listeria monocytogenes* (21), *Chlamydia trachomatis* (22), and *Mycobacterium tuberculosis* (23). Nonetheless, Dr. Eissa’s group using autophagy-related gene (ATG)-deficient mice revealed that deficiency of autophagy enhanced phagocytosis of invading microbes through the upregulation of phagocytic receptors (24), suggesting that autophagy may be an intrinsic negative feedback loop to control the excessive activation of macrophages. In regard to PA infection, it has been shown that this kind of extracellular bacterium can induce autophagy in both mast cells (25) and macrophages (26). We have previously shown that PA infection induced autophagy in macrophages, in return, the induction of autophagy inhibited PA internalization by downregulating the expression of phagocytic receptors and suppressed intracellular killing of engulfed PA *via* the reduction of reactive oxygen species (ROS) and reactive nitrogen species (RNS) production (27). This may be one of the immune evasion strategies that is used by PA.

In the present study, we explored a novel mechanism by which BD2 and BD3 promote bacterial clearance of PA in macrophages. Our results demonstrate that BD2 and BD3 inhibited the expression and nuclear translocation of early growth response gene-1 (EGR1) and proto-oncogene c-FOS and, therefore, promoted phagocytosis and intracellular killing of PA by inhibiting macrophage autophagy. These findings may provide a potential therapeutic approach and target for bacterial infections.

## Materials and Methods

### Materials and Reagents

*Pseudomonas aeruginosa* (strain 19660, American Type Culture Collection, Manassas, VA, USA) was grown in *Pseudomonas* isolation agar (REF 292710, Difco, BD, Sparks, MD, USA). Primary antibody against microtubule associated protein 1 light chain 3 (LC3) (REF NB910-40435, Novus Biologicals, Littleton, CO, USA), anti-β-actin (REF A1978, clone AC-15, Sigma-Aldrich, St. Louis, MO, USA), anti-Lamin B (REF sc-6216, clone B-10, Santa Cruz Biotechnology, CA, USA), anti-human beta-Defensin 3 (REF AE1050, clone L3-18b-E1, Immundiagnostik), anti-human beta-Defensin 2 (REF AE1110, clone L12-4C-C2, Immundiagnostik, Produktion, Germany), anti-c-FOS (REF sc-52, clone E-4, Santa Cruz Biotechnology, CA, USA), and anti-EGR1 (REF sc-189, clone C-19, Santa Cruz Biotechnology, CA, USA) were used in this study. Recombinant Human BD2 (REF 300-49) and BD3 (REF 300-52) were purchased from Prepotech (Rocky Hill, CT, USA). Phorbol 12-myristate 13-acetate (PMA, REF P1585), protease Inhibitor Cocktail (REF P8340), DTT (REF DTT-RO), phenylmethylsulfonyl fluoride (REF PMSF-RO), bovine serum albumin (BSA) (REF B2064), and gentamicin (REF E003632) were purchased from Sigma-Aldrich (St. Louis, MO, USA). Zymosan Alexa Fluor 488 Fluorescent Bioparticles (REF Z23373), DAPI (REF D1306), trizol (REF 10296028), fetal bovine serum (FBS, 10099141), penicillin–streptomycin (REF15140122), RPMI 1640 Medium (REF 31870082), DMEM medium (REF 11965118), RevertAid First Strand cDNA synthesis kit (REF K1622), SYBR Green Master Mix (REF A25743), Lipofectamine™ 2000 (REF 11668019), DQ-Red BSA (REF L7528), and Filmtracer Green Biofilm (FTGB, REF F10317) were purchased from Thermo Fisher Scientific (Waltham, MA, USA). Affymetrix GeneChip was conducted and analyzed by CapitalBio Corporation (Beijing, China).

### Cell Culture

Human monocytic cell line THP-1 (ATCC, TIB-202, Rockville, MA, USA) were treated with PMA (80 nM) at 37°C for 16 h, aiming to obtain the PMA-differentiated THP-1 macrophages. THP-1 cells were cultured in RPMI 1640 medium supplemented with 10% FBS and 1% penicillin–streptomycin. Murine macrophage-like cell line RAW264.7 (ATCC, TIB-71) was cultured in DMEM medium supplemented with 10% FBS, 1% penicillin–streptomycin, and 1% l-glutamine. Cells were then incubated at 37°C in a humidified incubator with 5% CO_2_.

### Real-time (RT) PCR

Real-time PCR was utilized to characterize the mRNA expression of different genes. First, total RNA was extracted from cell pellets using Trizol, and then 1 µg of total RNA was reversely transcribed to cDNA by using RevertAid First Strand cDNA synthesis kit. Next, the cDNA was amplified using SYBR Green Master Mix with the primers listed in Table S1 in Supplementary Material. The quantitative RT PCRs were conducted using the CFX96 RT PCR System (Bio-Rad, Hercules, CA, USA). Finally, the relative expression of each target gene was calculated after normalization to the level of β-actin mRNA. The primers for constructing expression pSG5 vectors containing *EGR1* or *c-FOS* genes are also listed in Table S1 in Supplementary Material.

### Transient Transfection

THP-1 and RAW264.7 cell lines were transiently transfected with scrambled negative control siRNA (sc-37007) vs siRNAs (purchased from Santa Cruz Biotechnology) against BD2 (sc-43722), BD3 (sc-40483), c-FOS (sc-29222), and EGR1 (sc-35267), or with the control pSG5 vector vs pSG5-EGR1 and pSG5-c-FOS expression vectors, using Lipofectamine™ 2000 according to the manufacturer’s instructions.

### Western Blot

Cells were rinsed three times with ice-cold phosphate buffered saline (PBS, pH 7.4) and treated with lysis buffer containing 1% (v/v) protease inhibitor cocktail, 1 mM phenylmethylsulfonyl fluoride, and 1 mM DTT. Cell lysates with equivalent protein amounts (20 µg) were loaded, thereafter separated by SDS-PAGE, and then transferred to poly (vinylidene difluoride) membrane. The membranes were blocked in PBS-Tween20 (pH 7.4, 0.5% Tween20) with 5% BSA, and then incubated overnight with the primary antibodies at 4°C. Then, the membranes were incubated with appropriate horseradish peroxidase-conjugated secondary antibodies at room temperature (RT) for 1 h, and at last visualized with an ECL kit (KeyGEN, Nanjing, China) according to the manufacturer’ instruction.

### Immunofluorescence Staining and Confocal Microscopy

THP-1 macrophages and RAW264.7 cells were seeded on the coverslips pre-coated with collagens in 24-well plates. After transfection with siRNA for 24 h or addition with the recombinant BD2 and BD3 (1 µg/ml) for 6 h, cells were infected with PA at a multiplicity of infection (MOI) 1 for 6 h. Next, cells were fixed with 4% paraformaldehyde, followed by membrane permeabilization using 0.2% Triton-X-100. Then, the coverslips were incubated in 5% BSA, and were sequentially incubated with primary LC3 antibody and secondary Alexa Fluor 488 goat anti-rabbit IgG Ab before mounting. Finally, the coverslips were observed under a ZEISS IMAGER A1 fluorescence microscope (CARL ZEISS) to capture fluorescence images. For confocal microscopy, cells were treated with siRNA or BD2/3 and DQ-red BSA (lysosome marker) for 6 h, and thereafter challenged with Zymosan Alexa Fluor 488 Fluorescent Bioparticles at MOI 25 for 1 h. After fixation and blockade, nuclei were then stained with the blue-fluorescent dye DAPI, and cells were visualized using a confocal microscope (Zeiss Axiovert, LSM710).

### Plate Count to Evaluate Phagocytosis and Intracellular Killing of PA

The efficiency of phagocytosis and intracellular bacterial killing was evaluated by bacterial colony count as described before (28). Cells were treated with recombinant BD peptides (1 µg/ml) for 6 h, or transfected with siRNAs (against BD2/3) or expression plasmid (including BD2/BD3/EGR1/c-FOS) for 24 h. Thereafter, cells were infected with PA at MOI 25. After 1 h of PA infection, gentamicin was added into the medium and incubated for 30 min to kill extracellular PA. Next, cells were washed with PBS for three times to remove the extracellular PA, and then lysed with 0.1% Triton-X. The numbers of phagocytized alive bacteria were determined by colony count assay. Cell lysates in a series of 10-fold dilutions were plated in triplicate on *Pseudomonas* isolation agar, and then incubated overnight at 37°C. The phagocytosis efficiencies of PA were identified as bacterial colony forming units (CFU) per cell at 1 h post-infection [CFU (1 h)]. In order to determine the efficacy of intracellular killing of PA, after removal of extracellular bacteria from cells at 1 h post-infection, cells were thereafter incubated for another hour and then lysed to plate. The survived bacteria at CFU per cell [CFU (2 h)] were calculated. The killing efficiencies of the internalized PA were determined using the following equation: Intracellular bacterial killing = [CFU (1 h) − CFU (2 h)]/CFU (1 h) × 100%.

### Phagocytosis Assay Assessed by Flow Cytometry

Phagocytosis measured by flow cytometry was performed as described previously (27). THP-1 macrophages were pretreated with BD2 and BD3 for 6 h, and after infected with FTGB-labeled PA at MOI 25. Following 1 h bacterial incubation, cells were washed three times utilizing cold PBS and centrifuged to remove the extracellular PA. Then THP-1 cells were analyzed using a Beckman Coulter EPICS XL/MCL (Beckman Coulter Inc., Fullerton, CA, USA).

### Microarray Analysis

THP-1 cells were treated with recombinant BD2 or BD3 peptides for 12 h. Total RNA was extracted using TRIzol and purified with mirVana miRNA Isolation Kit (Ambion, Austin, TX, USA). RNA integrity was determined by capillary electrophoresis using the RNA 6000 Nano Lab-on-a-Chip kit and Agilent Bioanalyzer 2100 (Agilent Technologies, Inc., Santa Clara, CA, USA). Higher yields of cDNA were labeled with a fluorescent dye (Cy5 and Cy3-dCTP) using CapitalBio cRNA amplification and labeling kit (CapitalBio, Beijing, China). The labeled cRNAs from mRNAs were purified and hybridized to Agilent Human lncRNA + mRNA Array V3.0 (Agilent). The labeled and purified microRNAs were hybridized with Agilent Human microRNA Microarray Release 19.0 according to the manufacturer’s instructions. Raw data were normalized by MAS 5.0 algorithm in Gene Spring Software 11.0 (Agilent Technologies, Inc.). Cluster analysis of gene expression profile showed the upregulated (red) or downregulated (green) genes after BD2 and BD3 stimulation in comparison with unstimulated control. All genes with expression variation in the range of Log 1.15-fold of control were clustered.

### Statistical Analysis

An unpaired two-tailed Student’s *t*-test was used to analyze the differences between two groups; and to compare the differences between three or more groups, analysis of variance was employed followed by Bonferroni’s post-test. Differences were considered to be statistically significant when the *p-*value was <0.05.

## Results

### Increased Expression of BD2 and BD3 in Macrophages upon PA Infection

**Figure 1:**
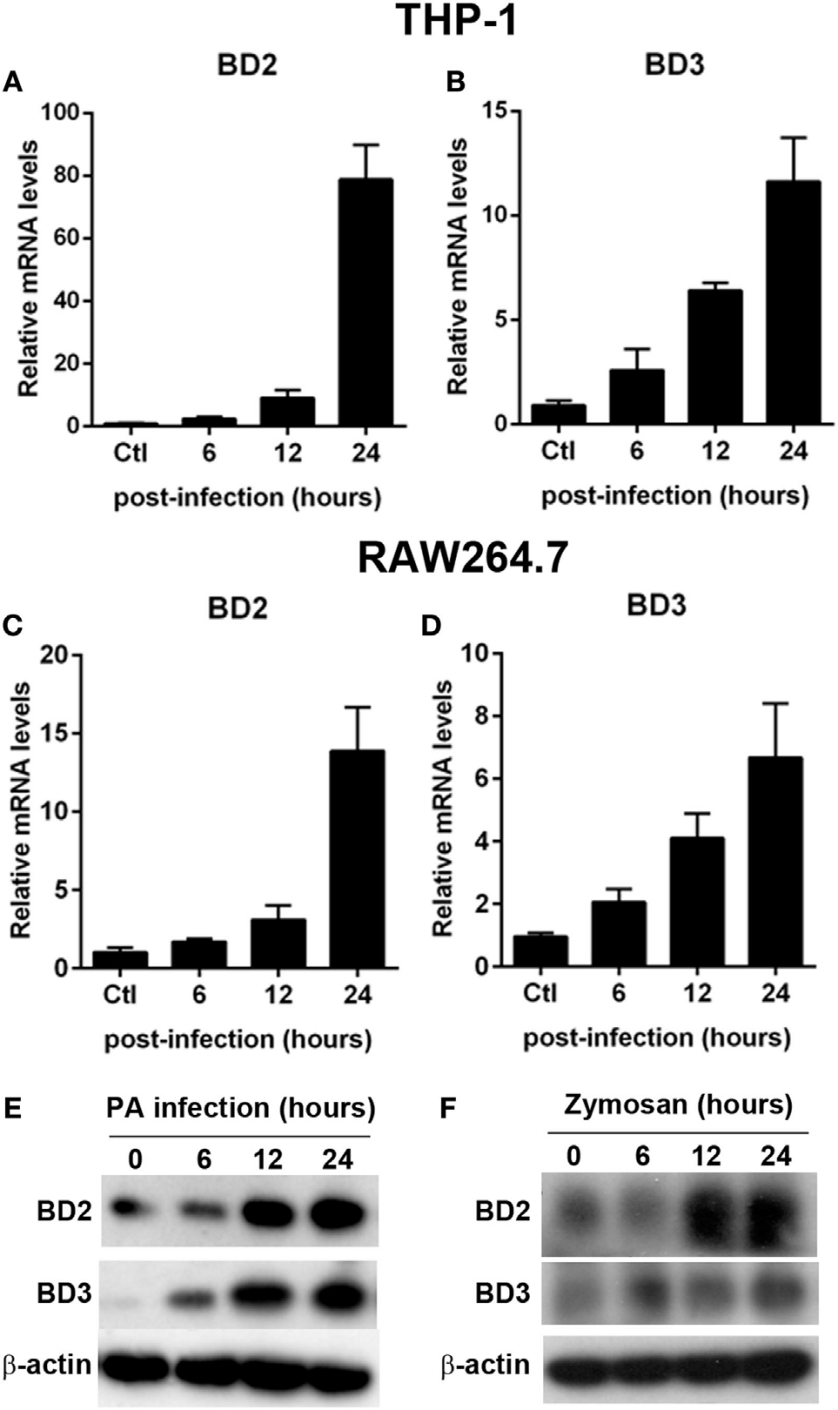
*Pseudomonas aeruginosa* (PA) infection increased beta-defensins 2 (BD2) and beta-defensins 3 (BD3) expression in macrophages. THP-1 macrophages **(A,B)** and RAW264.7 cells **(C,D)** were infected with PA at multiplicity of infection (MOI) 1. The mRNA levels of BD2 **(A,C)** and BD3 **(B,D)** were analyzed by real-time PCR. **(E)** THP-1 macrophages were infected with PA at MOI 1. Protein levels of BD2 and BD3 were tested by western blot. **(F)** THP-1 macrophages were incubated with Zymosan Bioparticles (MOI = 1) at indicated time. Protein levels of BD2 and BD3 were tested by western blot.

To determine whether BD2 and BD3 is expressed in macrophages upon PA infection, we analyzed the mRNA expression level of BD2 and BD3 in THP-1 differentiated human macrophages and murine macrophage-like RAW264.7 cells after PA infection. Data showed that BD2 and BD3 increased in THP-1 cells (Figures 1A,B) and RAW264.7 cells (Figures 1C,D). In addition, BD2 and BD3 protein expression upon PA infection were determined by western blot. Data showed that PA infection induced BD2 and BD3 protein levels on THP-1 cells in a time dependence (Figure 1E). Moreover, increased protein expression of BD2 and BD3 were observed in THP-1 cells upon Zymosan Bioparticles treatment (Figure 1F). These data indicated that PA infection induced BD2 and BD3 expression in macrophage.

### BD2 and BD3 Suppressed PA-Induced Autophagy in Macrophages

**Figure 2:**
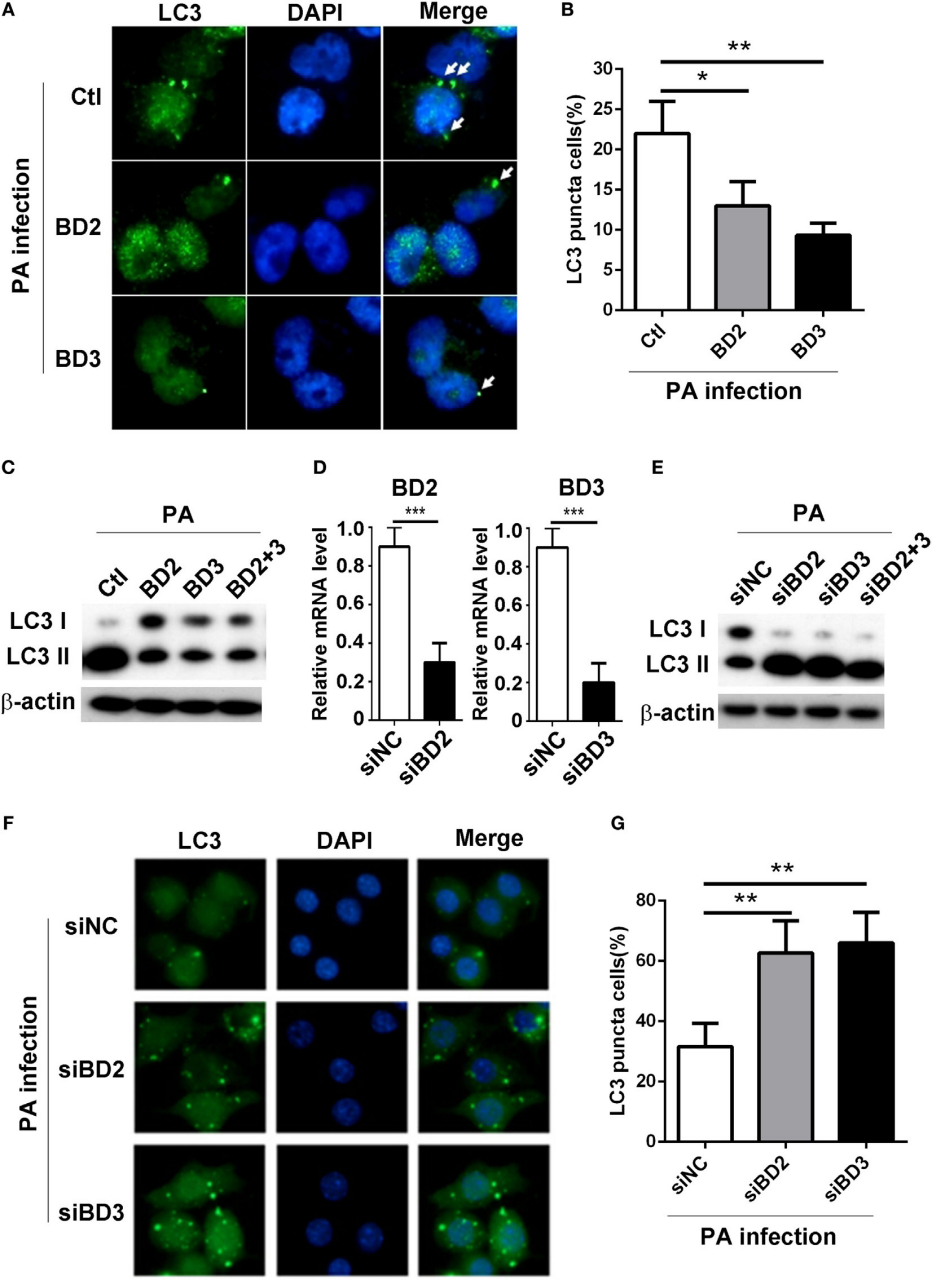
Beta-defensins 2 (BD2) and beta-defensins 3 (BD3) suppressed autophagy in macrophages. **(A–C)** THP-1 macrophages were treated with recombinant human BD2 or BD3 or both peptides (1 µg/ml) for 6 h, and infected with *Pseudomonas aeruginosa* (PA) at multiplicity of infection 1 for 6 h. **(D–G)** RAW264.7 cells were transiently transfected with siBD2, siBD3, or both vs siNC for 24 h, and then infected with PA. **(A,F)** Cells were fixed, stained with anti- microtubule associated protein 1 light chain 3 (LC3) Alexa Fluro 488 fluorescent Ab, and then detected by immune fluorescence microscopy. Arrows indicate the LC3 puncta in THP-1 macrophages **(A)** and RAW264.7 cells **(F)**. **(B,G)** Quantification of cells containing LC3 puncta in THP-1 macrophages **(B)** and RAW264.7 cells **(G)**. **(C,E)** Protein levels of LC3-II were tested by western blot in THP-1 macrophages **(C)** and RAW264.7 cells **(E)**. **(D)** The knockdown efficiency was tested by RT PCR. Data are shown as the mean ± SEM of three independent experiments (*p < 0.05; **p < 0.01; ***p < 0.001).

To explore whether BD2 and BD3 play a role on PA-induced autophagy in macrophages, THP-1 differentiated human macrophages were treated with recombinant peptides of BD2 or BD3 or both for 6 h, thereafter infected with PA strain ATCC 19660 at MOI 1 for 6 h. The formation of LC3 puncta was detected by immunofluorescent staining, and the conversion of LC3-I to LC3-II was assessed by western blot. Data showed that BD2 and BD3 peptides significantly decreased the percentage of cells containing LC3 puncta (Figures 2A,B) and repressed conversion of LC3-I to LC3-II in THP-1 macrophages (Figure 2C). To further confirm the role of BD2 and BD3 on PA-induced autophagy, murine macrophage-like RAW264.7 cells were transfected with siBD2 or siBD3 or both for 24 h, followed by PA infection for 6 h. The formation of LC3 puncta, and LC3-II protein expression was determined by immunofluorescent staining and western blot. Knockdown efficiency was confirmed by RT PCR (Figure 2D). *In vitro* knockdown of BD2 and BD3 macrophages promoted the conversion of LC3-I to LC3-II in RAW264.7 cells (Figure 2E) and increased percentage of cells with LC3 puncta (Figures 2F,G) when compared with the control group. Taken together, these results demonstrate that BD2 and BD3 participate in the suppression of PA-induced autophagy in macrophages.

### BD2 and BD3 Promoted Macrophage-Mediated Phagocytosis, Intracellular Killing of PA, and Internalization of Zymosan Bioparticles

**Figure 3:**
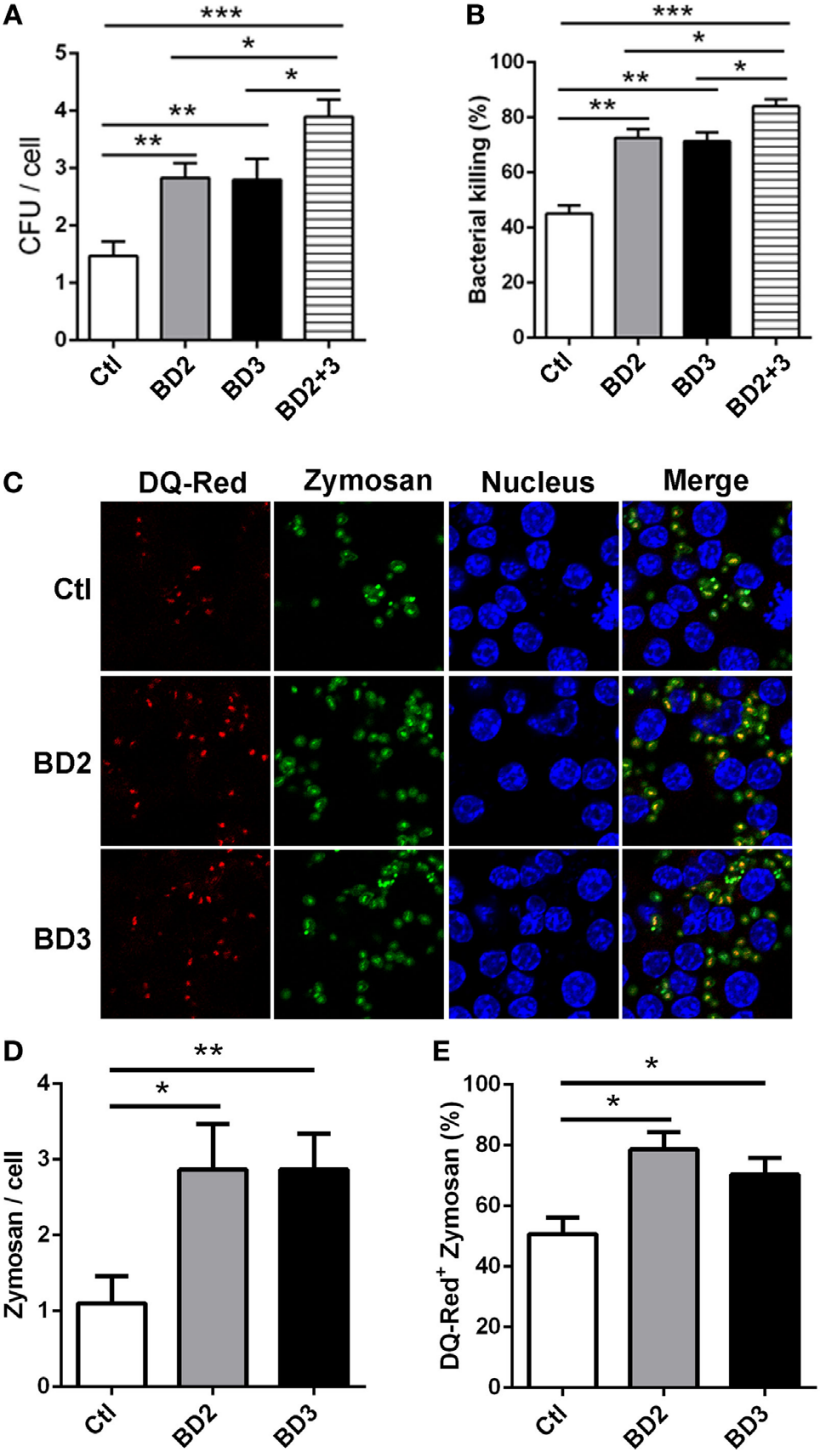
Beta-defensins 2 (BD2) and beta-defensins 3 (BD3) enhanced macrophage-mediated phagocytosis and intracellular killing of *Pseudomonas aeruginosa* (PA), and internalization of Zymosan bioparticles. **(A,B)** THP-1 macrophages were treated with recombinant BD2 or BD3 or both peptide (1 µg/ml) for 6 h, and infected with PA at multiplicity of infection (MOI) 25, and then the efficiency of phagocytosis **(A)** and intracellular killing **(B)** was determined by colony forming unit assay. **(C–E)** THP-1 macrophages were treated with BD2 or BD3 peptides (1 µg/ml) for 24 h, and treated with DQ-Red bovine serum albumin (lysosome marker, Red) for 6 h, and then incubated with Zymosan Alexa Fluro 488 Fluorescent Bioparticles (MOI = 25) for 1 h. Cells were fixed, stained with DAPI (Blue) to visualize the nuclei, and then examined by confocal microscopy [**(C)** scale bar = 5 µm]. Internalization of Zymosan bioparticles was calculated by the number of phagosomes containing fluorescent Zymosan bioparticles (green dots) per cell **(D)**. Formation of phagolysosomes was determined by the co-localization of DQ-red positive lysosomes (red) and phagosomes containing zymosan bioparticles (green) **(E)**. Data are shown as the mean ± SEM of three independent experiments (*p < 0.05; **p < 0.01; ***p < 0.001).

To address whether BD2 and BD3 are involved in regulating phagocytosis and macrophage-mediated PA eradication, THP-1 macrophages were treated with exogenous BD2, BD3 peptide or both for 6 h, thereafter infected with PA. The cell host phagocytosis and killing efficiency were determined by plate count assay. Interestingly, THP-1 macrophages treated with BD2 and BD3 displayed higher efficacy of PA phagocytosis and intracellular killing when compared to the untreated cells (Figures 3A,B). Compared with either BD2 or BD3 alone, administration of both peptides led to enhanced PA phagocytosis and intracellular killing (Figures 3A,B). To further confirm the possible effects of BD2 and BD3 on PA phagocytosis, we used the flow cytometry analysis. After incubation with BD2 or BD3 for 6 h, THP-1 cells were infected by the FTGB-labeled PA for 1 h, and the phagocytosis efficiency was assessed. We found that the internalization percentages of PA were raised to 30.6 and 33.3% in THP-1 macrophages pretreated with BD2 or BD3 peptides, respectively; which were higher than the unstimulated control (14.8%) (Figure S1 in Supplementary Material). Next, we questioned whether BD2 and BD3 regulated macrophage-mediated phagocytosis of other particles. THP-1 macrophages pretreated with BD2 and BD3were stimulated with fluorescently labeled zymosan bioparticles at an MOI 25 for 1 h, and then stained with DQ-red, a fluorogenic substrate that identifies proteases located in lysosomes. Our confocal microscopy data revealed an increased number of phagosomes containing zymosan bioparticles after treatment with BD2 or BD3 (Figures 3C,D). In addition, the co-localization of DQ-red positive lysosomes (red) and phagosomes containing zymosan bioparticles (green) was increased in THP-1 macrophages treated with BD2 or BD3 (Figures 3C,E), suggesting that induction of autophagy decreased formation of phagolysosomes associated with zymosan bioparticles. Together, these data indicate that BD2 and BD3 promote macrophage-mediated phagocytosis, intracellular killing of PA, and internalization of Zymosan bioparticles.

### Silencing of BD2 and BD3 Suppressed Macrophage-Mediated Phagocytosis, Intracellular Killing of PA, and Internalization of Zymosan Bioparticles

**Figure 4:**
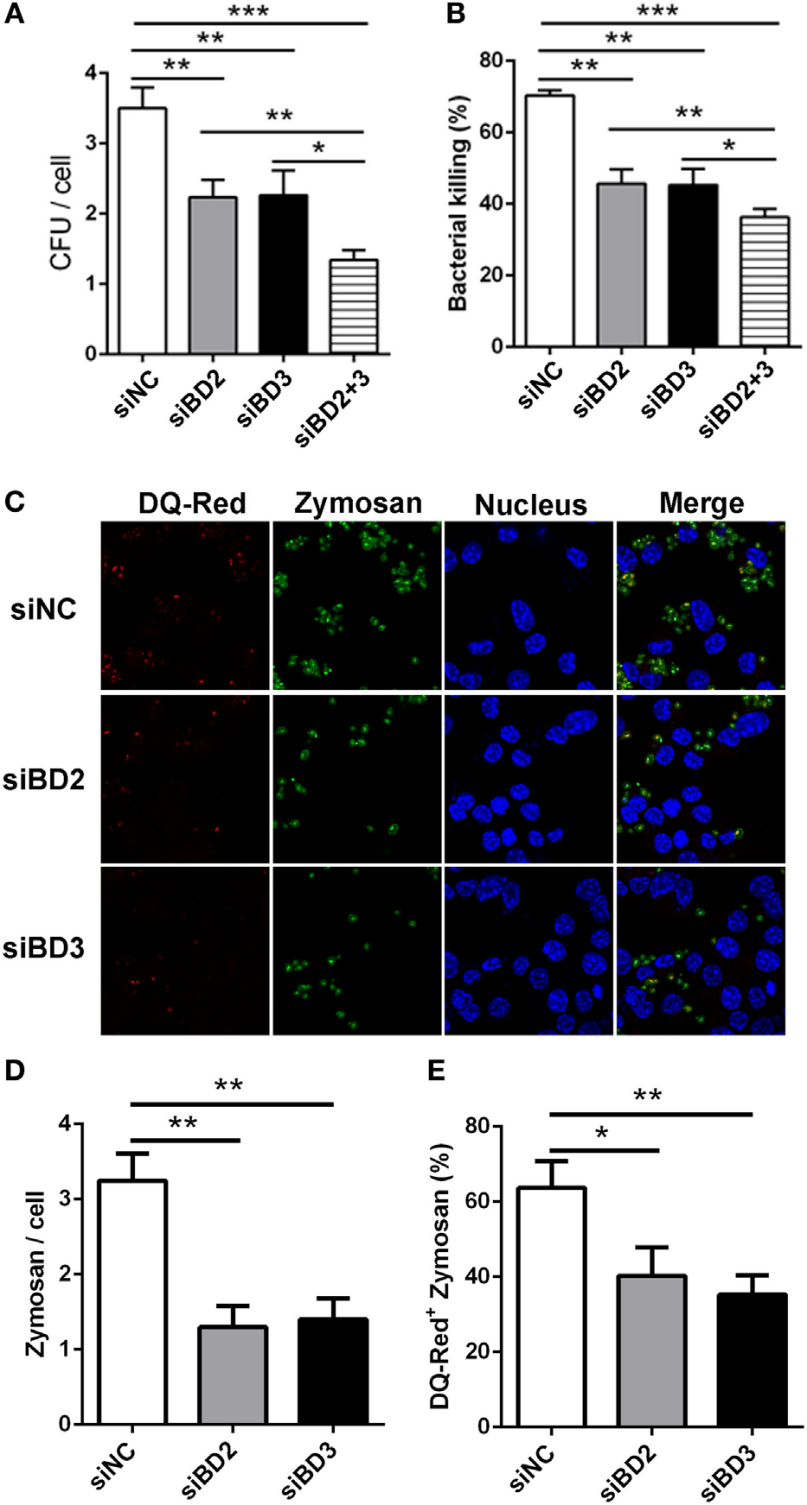
Silencing of beta-defensins 2 (BD2) and beta-defensins 3 (BD3) decreased macrophage-mediated phagocytosis, bacterial killing of *Pseudomonas aeruginosa* (PA), and internalization of Zymosan bioparticles. **(A,B)** RAW264.7 cells were transfected with siBD2 or siBD3 or both for 24 h, and infected with PA at multiplicity of infection (MOI) 25 for 1 or 2 h, and then accessed by phagocytosis or intracellular killing with plate count assay. **(C)** After siRNA transfection, RAW264.7 cells were treated with DQ-Red bovine serum albumin (lysosome marker, Red) for 6 h, and then incubated with Zymosan Alexa Fluro 488 Fluorescent Bioparticles (MOI = 25) for 1 h. Cells were fixed, stained with DAPI (Blue) to visualize the nuclei, and then examined by confocal microscopy (scale bar = 5 µm). **(D)** Internalization of Zymosan bioparticles was calculated by the number of phagosomes containing fluorescent Zymosan bioparticles (green dots) per cell. **(E)** Formation of phagolysosomes was determined by the co-localization of DQ-red positive lysosomes (red) and phagosomes containing Zymosan bioparticles (green) in RAW264.7 cells. Data are shown as the mean ± SEM of three independent experiments (*p < 0.05; **p < 0.01; ***p < 0.001).

To further assess the role of BD2 and BD3 on macrophage-mediated phagocytosis, intracellular killing of PA, and internalization of Zymosan bioparticles, RAW264.7 macrophages were transiently transfected with siBD2 or siBD3 for 24 h, and was then followed by PA infection. Plate count assays demonstrated that the knockdown of BD2 or BD3 significantly reduced the phagocytosis capacity (Figure 4A) and intracellular killing efficiency (Figure 4B) of PA in RAW264.7 cells, when compared to the negative control group. When compared with knockdown of either BD2 or BD3 alone, silencing both defensins led to reduced PA phagocytosis and intracellular killing (Figures 4A,B). Additionally, transfection with siBD2 or siBD3 decreased the internalization of Zymosan bioparticles (Figures 4C,D) and the formed phagolysosomes containing Zymosan bioparticles (Figures 4C,E) in RAW264.7 cells. Collectively, these results indicate that silencing of BD2 and BD3 leads to suppression of macrophage-mediated phagocytosis, intracellular killing of PA, and internalization of Zymosan bioparticles.

### BD2 and BD3 Promoted Macrophage-Mediated Phagocytosis and Intracellular Bacterial Killing by Inhibiting Autophagy

**Figure 5:**
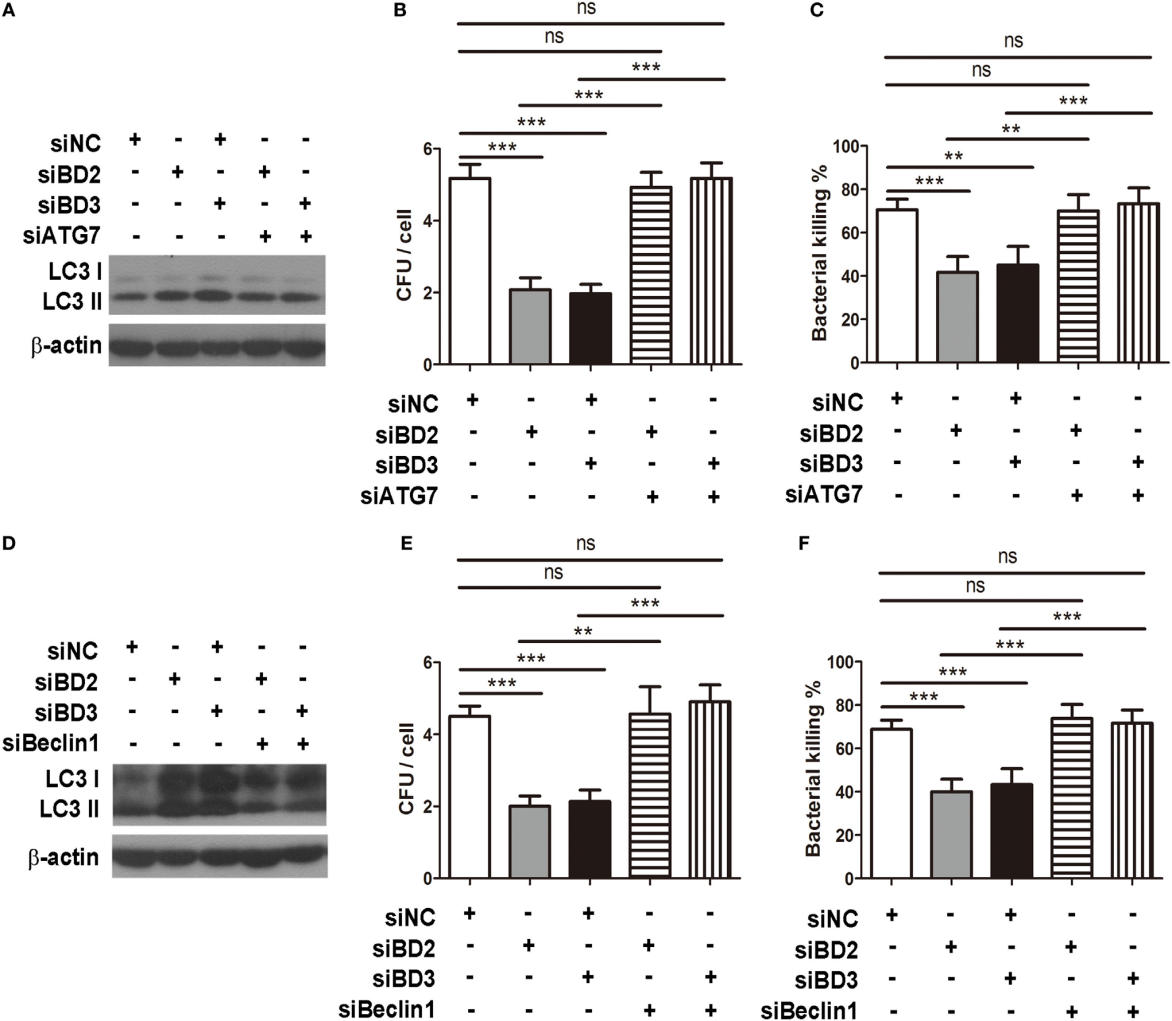
Beta-defensins 2 (BD2) and beta-defensins 3 (BD3) promoted macrophage-mediated phagocytosis and intracellular killing of *Pseudomonas aeruginosa* (PA) by inhibiting autophagy. RAW264.7 cells were transfected with siBD2, siBD3, siBecin1, or siATG7 for 24 h, and infected with PA at multiplicity of infection 25 for 1 or 2 h. Microtubule-associated protein 1 light chain 3 (LC3)-II protein expression was determined by western blot **(A,D)**. Phagocytosis **(B,E)** and intracellular killing **(C,F)** was accessed by plate count assay. Data are shown as the mean ± SEM of three independent experiments (**p < 0.01; ***p < 0.001).

To investigate whether BD2 and BD3 enhance phagocytosis and intracellular killing of PA in macrophage through downregulation of autophagy, RAW264.7 macrophages were transiently transfected with siBD2, siBD3, siBeclin1, or siATG7 for 24 h, and the transfection was followed by PA infection. Knockdown of autophagy-related protein Beclin1 with siRNA attenuated LC3-II expression induced by siBD2 or siBD3 transfection (Figure 5A). In addition, knockdown of Beclin1 restored phagocytosis (Figure 5B) and intracellular killing of PA (Figure 5C) suppressed by siBD2 or siBD3 transfection. Knockdown of autophagosome formation associated protein ATG7 decreased LC3-II protein levels induced by silencing BD2 or BD3 (Figure 5D). Knockdown of ATG7 restored phagocytosis (Figure 5E) and intracellular killing of PA (Figure 5F) suppressed by the silence of BD2 or BD3. This finding shows that BD2 and BD3 promote macrophage-mediated phagocytosis and intracellular killing of PA by inhibiting autophagy.

### BD2 and BD3 Inhibited the Expression and Nuclear Translocation of EGR1 and c-FOS

**Figure 6:**
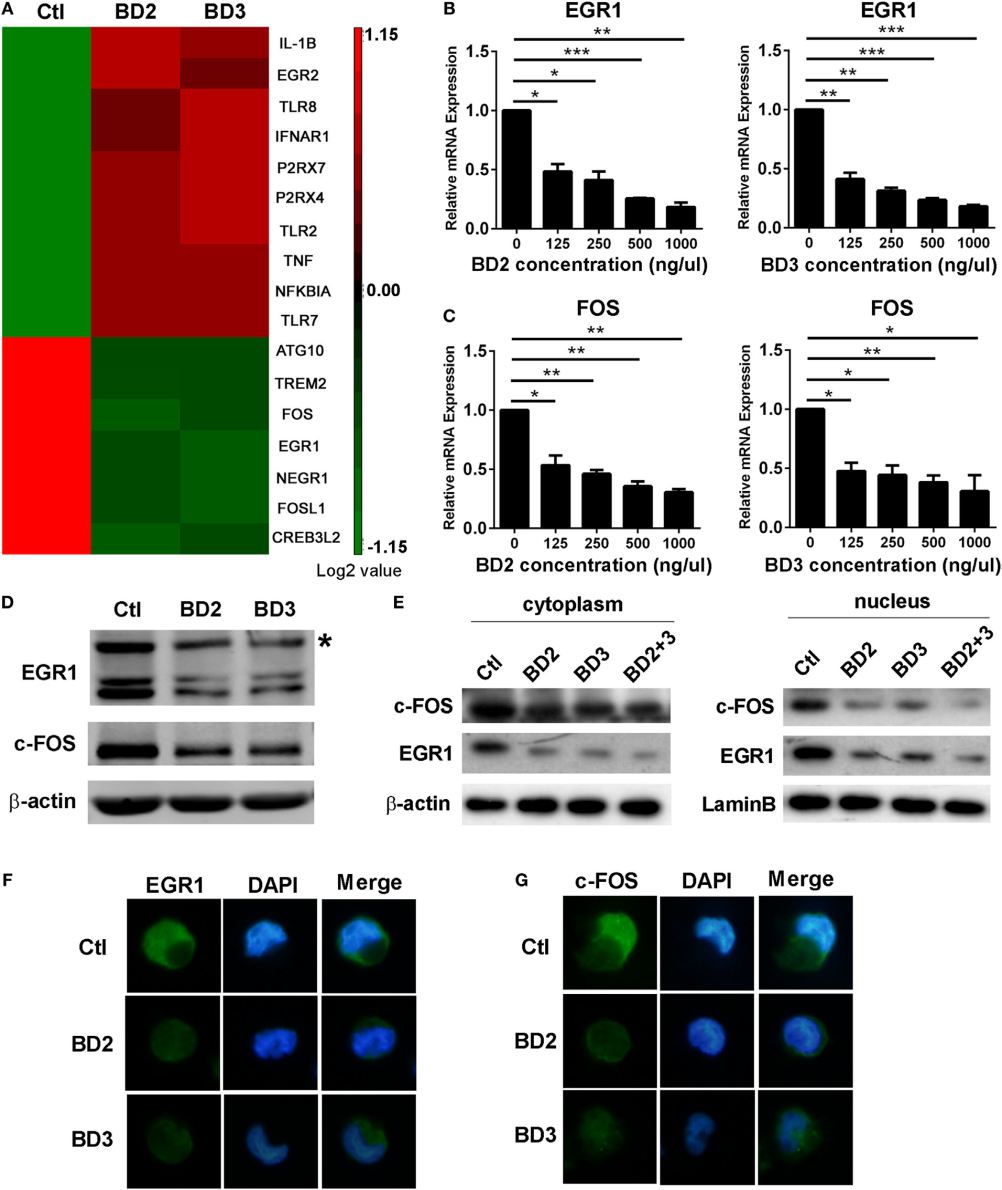
Beta-defensins 2 (BD2) and beta-defensins 3 (BD3) reduced early growth response gene-1 (EGR1) and c-FOS expression and nuclear translocation in macrophages. **(A)** Affymetrix GeneChip was used to detect mRNA expression profiles in THP-1 macrophages stimulated with recombinant BD2 or BD3 peptides. Cluster analysis of gene expression profile showed the upregulated (red) or downregulated (green) genes after BD2 and BD3 stimulation in comparison with unstimulated control. All genes with expression variation in the range of 1.15-fold of control were clustered. The mRNA levels of EGR1 **(B)** and c-FOS **(C)** were tested by RT PCR in THP-1 macrophages after treatment with recombinant human BD2 or BD3 peptides at the indicated concentrations. Protein levels of EGR1 and c-FOS in the whole cell lysate **(D)** or in the separated part of cell cytoplasm (left) or nuclei (right) **(E)** were examined by western blot. Target band of c-FOS (62kD) was labeled with asterisk (D). **(F,G)** THP-1 cells plated in coverslips were treated with BD2 or BD3 peptides, following by PA infection. Then, the coverslips were sequentially incubated with primary EGR1 or c-FOS antibody and secondary Alexa Fluor 488 goat anti-rabbit IgG Ab. Finally, the coverslips were observed under a fluorescence microscope to capture fluorescence images. Data are shown as the mean ± SEM of three independent experiments (*p < 0.05; **p < 0.01; ***p < 0.001).

Microarray analysis of THP-1 macrophages stimulated with BD2 or BD3 showed that the expression levels of some genes, including autophagy-related 10 (ATG10), EGR1, and fos proto-oncogene FOS, were downregulated under BD2 or BD3 treatment (Figure 6A). We further confirmed these genes by RT PCR, and data show that BD2 and BD3, at different concentrations in the range from 125 to 1,000 ng/µl, significantly inhibited the RNA expression of EGR1 (Figure 6B) and c-FOS (Figure 6C). Western blot results showed that the expression level of EGR1 and c-FOS proteins decreased in macrophages stimulated with BD2 or BD3 (Figure 6D). In addition, the expression levels of these proteins were reduced in the nuclei and cytoplasm (Figure 6E), suggesting that BD2 and BD3 inhibited the translocation of EGR1 and c-FOS into the nuclei. Furthermore, immunofluorescence showed that administration of BD2 or BD3 inhibited the translocation of EGR1 (Figure 6F) and c-FOS (Figure 6G) into nuclei. These results show that BD2 and BD3 inhibit the expression and nuclear translocation of EGR1 and c-FOS.

### EGR1 and c-FOS Enhanced Autophagy in Macrophages

**Figure 7:**
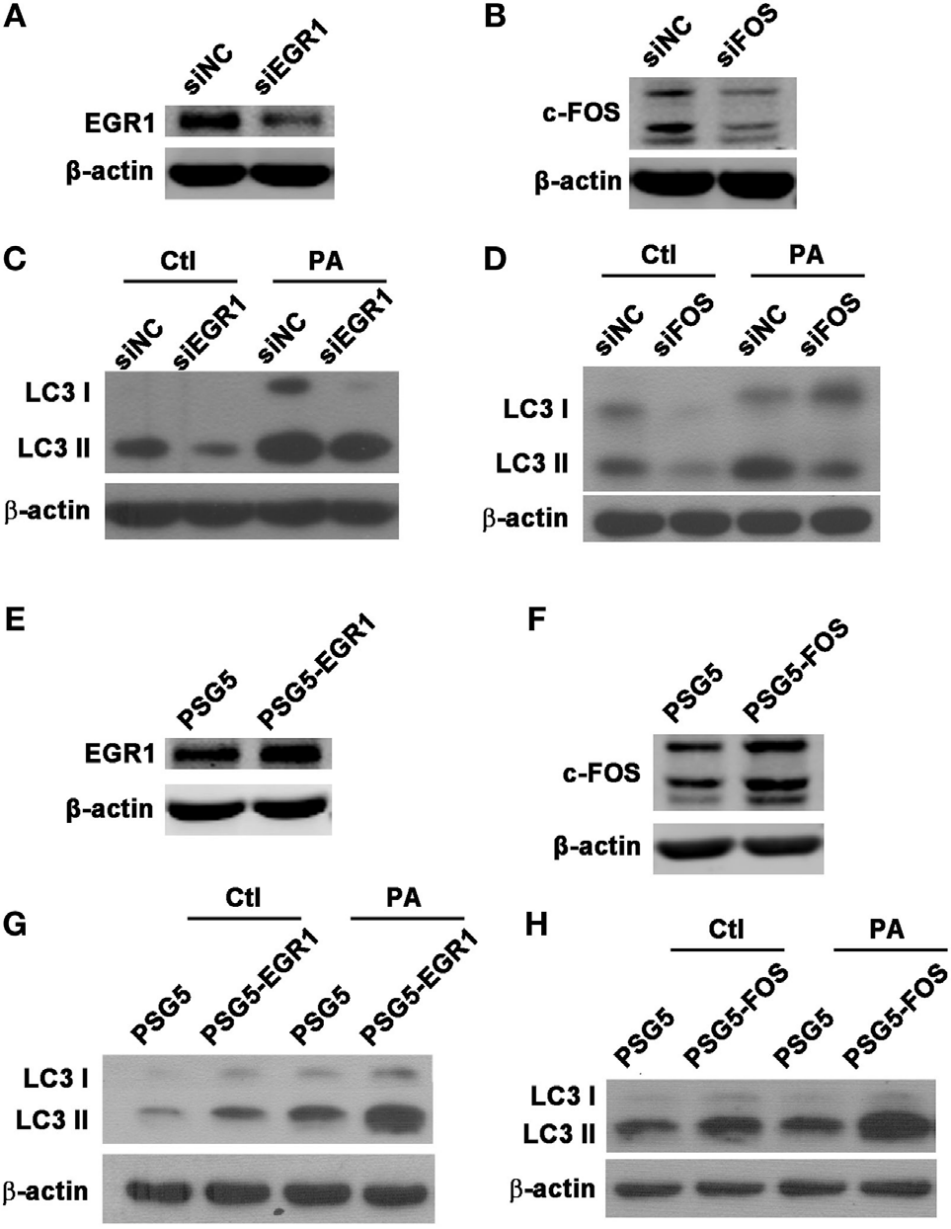
Early growth response gene-1 (EGR1) or c-FOS enhanced autophagy in macrophages. **(A–H)** THP-1 macrophages were transfected with specific siRNA or overexpression plasmid for EGR1 or c-FOS for 24 h, and then infected with *Pseudomonas aeruginosa* (PA) for 1 h. Knockdown and overexpression effects of EGR1 **(A,E)** and c-FOS **(B,F)** were determined by western blot. Protein levels of microtubule associated protein 1 light chain 3 (LC3)-II **(C,D,G,H)** were tested by western blot in THP-1 macrophages before or after PA infection.

To explore whether EGR1 and c-FOS modulate autophagy in macrophages during PA infection, the protein expression of autophagy-associated LC3-I and LC3-II were identified using western blot following the knockdown or overexpression of EGR1 and c-FOS in macrophages. The results showed silenced EGR1 (Figure 7A) and c-FOS (Figure 7B) reduced the LC3-II expression (Figures 7C,D) induced by PA infection in THP-1 macrophage. Overexpressed EGR1 (Figure 7E) and c-FOS (Figure 7F) enhanced the LC3-II expression (Figures 7G,H) in PA infected THP-1 macrophage compared with vector control. Taken together, these results demonstrate that EGR1 and c-FOS promote autophagy in macrophages during PA infection.

### Silencing of EGR1 or c-FOS Enhanced the Expression of Phagocytic Receptors

**Figure 8:**
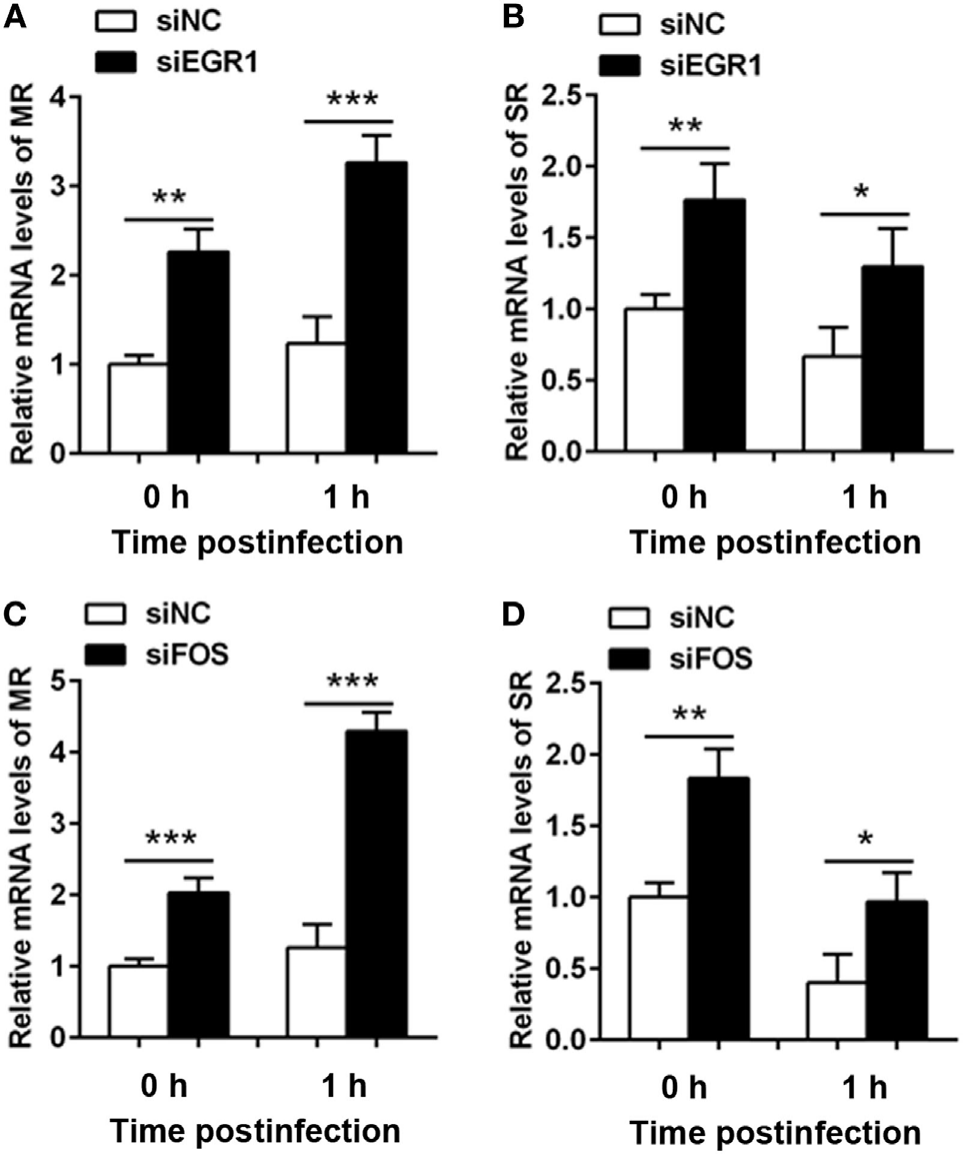
Silencing of early growth response gene-1 (EGR1) or c-FOS increased the expression of phagocytic receptors. THP-1 macrophages were transfected with siEGR1 **(A,B)** or siFOS **(C,D)** for 24 h, and then infected with *Pseudomonas aeruginosa* at multiplicity of infection 1 for 1 h. The mRNA levels of phagocytic receptors including mannose receptor (MR) **(A,C)** and scavenger receptor (SR) **(B,D)** were analyzed by real-time PCR. Data are shown as the mean ± SEM of three independent experiments (*p < 0.05; **p < 0.01; ***p < 0.001).

Next, we investigated the effect of EGR1 and c-FOS on the phagocytosis, the RNA levels of phagocytic receptors mannose receptor (MR) and scavenger receptor (SR) were determined in THP-1 macrophage cells after silencing EGR1 and c-FOS for 24 h, and followed by PA infection for 1 h. EGR1 and c-FOS knockdown significantly increased the RNA expression of MR (Figures 8A,C) and SR (Figures 8B,D) compared with the control siNC before and after PA infection, indicating that EGR1 and c-FOS inhibited the expression of phagocytic receptors.

### EGR1 or c-FOS Reversed the Effects of BD2 and BD3 on Phagocytosis and Intracellular PA Killing

**Figure 9:**
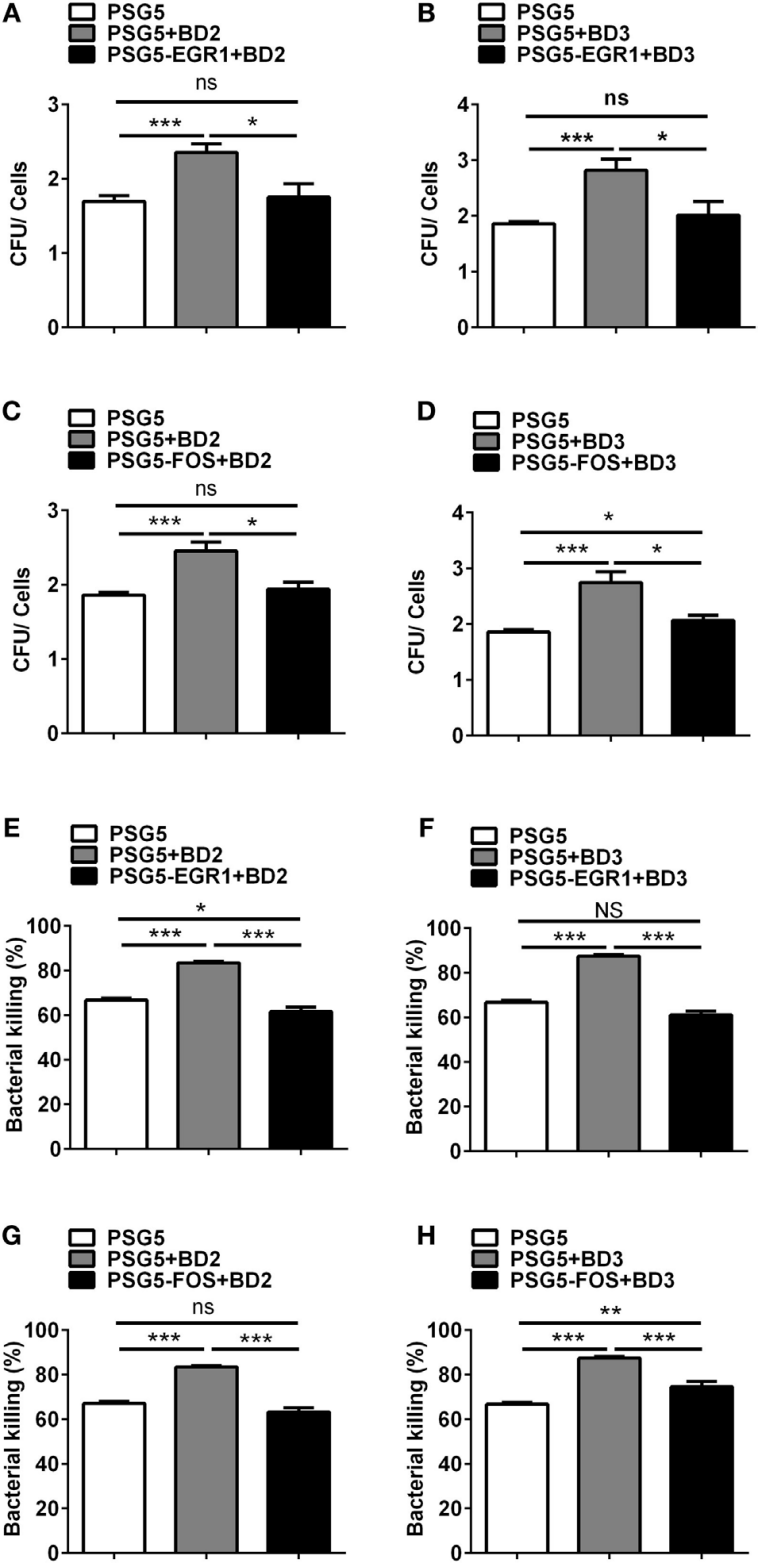
Overexpression of early growth response gene-1 (EGR1) or c-FOS reversed the role of beta-defensins 2 (BD2) and beta-defensins 3 (BD3) in macrophage-mediated *Pseudomonas aeruginosa* (PA) elimination. THP-1 cells were transfected with EGR1, c-FOS, BD2, or BD3 overexpression plasmid control vs pSG5 plasmid for 24 h, followed by PA infection at multiplicity of infection 25, and then analyzed by phagocytosis **(A–D)** and killing assay **(E–H)** using the plate count method. Data are shown as the mean ± SEM of three independent experiments (*p < 0.05; **p < 0.01; ***p < 0.001).

To illustrate the effects of EGR1 or c-FOS on the regulation of BD2 and BD3-mediated clearance of PA, THP-1 macrophages were transfected with EGR1 or c-FOS plasmids, then treated with BD2 or BD3 peptides for 6 h, which was followed by PA infection (MOI = 25). The efficiencies of phagocytosis and intracellular bacterial killing in THP-1 macrophages were determined by CFU array. Overexpressed EGR1 (Figures 9A,B) or c-FOS (Figures 9C,D) significantly reduced the efficacy of BD2 or BD3 on phagocytosis of PA. Similarly, overexpressed EGR1 (Figures 9E,F) or c-FOS (Figures 9G,H) significantly reversed the intracellular killing of PA, which was enhanced by BD2 or BD3 in macrophages. These results indicate that BD2 and BD3 promote macrophage-mediated clearance of PA through downregulation of EGR1 and c-FOS.

## Discussion

As a class of AMPs, BDs display broad-spectrum antimicrobial activities. However, their direct bactericidal effects usually rely on a high concentration, which is hardly reached in physiological conditions. Recently, increased evidence showed that BDs, especially BD2 and BD3, which are induced by infection or inflammation, also function as an immune modulator in both innate and adaptive immunity (8, 17, 18). However, whether BD2 and BD3 participate in the immune modulation of other bacterial eradication processes remain unknown. In this study, we demonstrated for the first time that BD2 and BD3 promoted phagocytosis and intracellular killing of PA by inhibiting macrophage autophagy through downregulation of EGR1 and c-FOS.

Innate immune cells liked macrophages employ several defense mechanisms against invading pathogens. Phagocytosis and intracellular killing are two of the most important steps for bacterial eradication (29–31). Phagocytosis is the first step of bacterial clearance, which means the internalization of bacteria outside the phagocytes. Whereas, intracellular killing is the second step, which means the lysis or degradation of phagocytosed bacteria. The intracellular killing system involves both oxygen-dependent components (e.g., production of ROS and RNS), and oxygen independent components (e.g., production of AMPs and lysozymes) (32–34). Some evidences suggest that programmed cell death like apoptosis and autophagy may function as a host protective strategy against invading bacteria. In contrast, other studies reported that induction of autophagy can suppress the phagocytic capability of macrophages, and this may be an escape mechanism for a variety of bacteria, including PA (27, 35), *Mycobacterium tuberculosis* (24), *Staphylococcus aureus*, and *Escherichia coli* (24).

*In vitro* studies have reported that BD2 and BD3 enhance the expressions of pro-inflammatory cytokines and chemokines *via* a TLR-dependent manner (11, 15). While *in vivo* knockdown of BD2 and BD3 enhanced inflammatory response, but reduced bacterial clearance in PA-infected mouse corneas (17, 18), indicating that BD2 and BD3 may regulate bacterial eradication through other mechanisms, rather than inflammatory modulation. Recent studies showed that the induction of autophagy suppresses macrophage-mediated phagocytosis and intracellular killing of PA (27, 35). However, the relationship between BD2 and BD3 in autophagy and phagocytosis remains undefined. In this study, we found that BD2 and BD3 strikingly reduced the conversion of LC3-I to LC3-II form and decreased the number of LC3 puncta in human and murine macrophages, suggesting that BD2 and BD3 suppress the PA-induced autophagy in macrophages. Meanwhile, BD2 and BD3 were also able to promote macrophage-mediated phagocytosis of Zymosan bioparticles and the formation of phagolysosomes containing the fluorescent Zymosan particle. The observation found here indicates that the enhanced role of BD2 or BD3 in bacterial phagocytosis and intracellular killing is, therefore, not a specific phenomenon limited to PA infection. Recently, a study has demonstrated that after internalization in the host cells, phagocytosed bacteria are intracellularly killed through oxygen-dependent or -independent mechanisms (32). Wang et al. reported that human BD3 induces ROS production in human airway smooth muscle cells *via* the activation of extracellular regulated MAP kinase (ERK), mitogen-activated protein kinase (MAPK) 8 (JNK), and nuclear factor kappa B (NF-κB) pathway (36). They also showed that BD2 and BD3 may activate MAPK or NF-κB pathway to induce ROS generation. In contrast, other studies using LPS to stimulate rat hepatocytes have demonstrated that rapamycin, the pharmacological agonist of autophagy, decreased production of ROS and RNS, as well as NF-κB activation (37). Our previous study also showed that induction of autophagy by rapamycin reduced the production of ROS and RNS, while restraint of autophagy by knockdown of ATG7 and Beclin1 enhanced ROS and RNS productions in mouse macrophages (27). Taking together, we speculated that BD2 and BD3 could enhance NO and ROS generation to promote intracellular killing of PA in macrophages, partially through suppressing the autophagy.

In phagocytosis and bacterial killing assay, THP-1 or RAW264.7 cells were cultured in a six-well plate and infected with PA at MOI 25 for 1 h. After, cells in one of the duplicate wells were treated with gentamycin at 300 mg/ml for 30 min and washed with PBS for three times to kill and remove the extracellular bacteria, and then lysed with 0.1% Triton-X. Cells in the other of the duplicate wells were incubated for another 1 h and lysed in the same way. Intracellular bacterial killing = [CFU (1 h) − CFU (2 h)]/CFU (1 h) × 100%. Since the infection time is very short (1 h), a high MOI is required to increase the number of internalized bacteria, as indicated by CFU (1 h). Because macrophages can kill most of the engulfed bacteria, it is hard to test the bacterial load of CFU (2 h) when using a low MOI infection. In other experiments, such as PCR, western blot, and immunofluorescence, THP-1 or RAW264.7 cells were infected with PA at MOI 1 for a long time (up to 24 h). If a high MOI (e.g., MOI 25) is used for a long time of infection, most cells will undergo cell death. In this regard, we choose different MOIs for different infection time, to ensure that most cells are alive and responding well to other challenges in these assays, according to other reports (28) and our previous study (35).

Autophagy is a highly conserved cellular event controlled by various regulatory signals, including AMP-activated protein kinase, B-cell leukemia/lymphoma-2 protein family members, mammalian target of rapamycin, and TGF-beta activated kinase 1/MAP3K7 binding protein 2/3 (38). During an infectious event, pattern recognition receptors such as TLRs (39, 40) and also other receptors such as nucleotide oligomerization domain-like receptors (35, 41) initiate and promote autophagy *via* pathogen associated molecular pattern induced immunologic pathways. BD2 is considered as a ligand for TLR4 (10), and TLR4 pathway is linked to autophagy in macrophages (40). Nevertheless, our results revealed that BD2 and BD3 inhibited autophagy in PA infected macrophages, which indicated that BD2 and BD3 inhibited autophagy independent of TLR4 pathway. Furthermore, we found that BD2 and BD3 reduced expression and nuclear translocation of EGR1 and c-FOS. This indicates that these two transcription factors may be downstream molecules of BD2 and BD3 signaling pathway. Egr-1 is a zinc finger transcription factor classified as an immediate-early response protein, which is reported to be induced by a wide variety of cellular stressors including growth factors, metabolites (e.g., glutamate) and neuronal activity (e.g., dopamine) through MAPK and PI3K/AKT pathways (42, 43). Fos proteins are regulated by various stimuli like cytokines and growth factors, dependent on MAPK pathways (44). Previous studies revealed that BD3 reduces the phosphorylation of p38 and c-Jun MAPK pathway in human umbilical vein endothelial cells (45). Bian and colleagues have recently found that BD3 is involved in the attenuation of the phosphorylation of p38 and ERK1/2 in MAPK pathway (13). Thus, we speculated that MAPK signaling may contribute in the BD2 and BD3-mediated suppression of EGR1 and c-FOS in macrophages. In addition, cystic fibrosis transmembrane conductance regulator(CFTR) (46) and cell membrane protein Annexin A2 (AnxA2) (47) in conjunction with autophagy have been previously reported to be indispensable for the control of PA infections. Our microarray and PCR data showed that BD2 and BD3 had little effects on the expression of these two genes (data not shown), indicating that CFTR and AnxA2 are not involved in the BD2 and BD3-mediated immune regulation of macrophage autophagy.

It is also worthwhile to mention that Egr-1 is implicated in the regulation of many genes, including angiogenic factors, cytokines, apoptotic factors, cell cycle factors, metabolic factors, and proteases (48, 49). Studies have reported that Egr-1 enhanced autophagy induced by cigarette smoke (50) or irradiation (51), by promoting autophagy gene LC3 expression. Moreover, c-FOS is one of leucine zipper protein FOS proteins that has been implicated in the regulation, proliferation, differentiation, and transformation of cells. C-FOS binds to Becn1/BECN1 promoter and enhances Beclin1 transcription to promote autophagy in response to the dopamine agonist (52). Our data showed that knockdown of EGR1 or c-FOS inhibited, while overexpression of EGR1 or c-FOS enhanced autophagy in PA-infected macrophages. This EGR1 and c-FOS-mediated regulation of autophagy in PA-infected macrophages may also through modulating the transcription of autophagic genes (e.g., LC3B and BECN1).

Furthermore, our results demonstrate that the knockdown of EGR1 or c-FOS increased the mRNA expression of SR and MR. SR and MR are both phagocytic receptors expressed in macrophages and responsible for PA internalization (53, 54). It has been demonstrated that autophagy could regulate the expression of phagocytic receptors in macrophages. Macrophages isolated from the Atg7 knockout vs control wild-type mice displayed an enhanced bacterial internalization of *Mycobacterium tuberculosis* by upregulating the expression of SR (24). The expression of SR, MR, complement receptors, and Fc receptors for IgG (FcγR) were shown to be decreased in RAW264.7 or THP-1 macrophages after autophagy induction by starvation or rapamycin (27). In this regard, we speculate that EGR1 or c-FOS reverse the BD2 and BD3-mediated regulation of phagocytosis, mainly though downregulation of SR and MR.

In summary, we have demonstrated a novel role and the underlying mechanism of BD2 and BD3-mediated immune regulation of phagocytosis, bacterial killing, and autophagy. BD2 and BD3 promote bacterial clearance of PA by inhibiting macrophage autophagy through downregulation of EGR1 and c-FOS. These findings broaden our knowledge of BDs, as well as the crosstalk between autophagy and bacterial clearance, and may be used as a potential therapeutic approach for bacterial infections.

## Author Contributions

MW, YoW, and NF wrote the manuscript. MW designed experiments. YoW, DL, YiW, XL, WQ, KC, and LF performed experiments and analyzed data. YZ and XH provided scientific expertise. MW supervised the project.

## Conflict of Interest Statement

The authors declare that the research was conducted in the absence of any commercial or financial relationships that could be construed as a potential conflict of interest.

## Abbreviations

PA, *Pseudomonas aeruginosa*; BD2, beta-defensins 2; BD3, beta-defensins 3; AMPs, antimicrobial peptides; LPS, lipopolysaccharide; TLRs, toll-like receptors; ATG, autophagy-related gene; ROS, reactive oxygen species; RNS, reactive nitrogen species; EGR1, early growth response gene-1; c-FOS, fos proto-oncogene; CCR6, C–C motif chemokine receptor 6; IL-6, interleukin 6; TNF, tumor necrosis factor; LC3, microtubule associated protein 1 light chain 3; MR, mannose receptor; SR, scavenger receptor; ERK, extracellular regulated MAP kinase; MAPK, mitogen-activated protein kinase; NF-κB, nuclear factor kappa B; AMPK, AMP-activated protein kinase; Bcl-2, B-cell leukemia/lymphoma-2; mTOR, mammalian target of rapamycin; TAB, TGF-beta activated kinase 1/MAP3K7 binding protein; PRRs, pattern recognition receptors; NLRs, nucleotide oligomerization domain (NOD)-like receptors; PAMPs, pathogen associated molecular pattern; CFTR, cystic fibrosis transmembrane conductance regulator; AnxA2, cell membrane protein Annexin A2; CR, complement receptors; FcγR, Fc receptors for IgG; CFU, colony forming units.
