# Supplementary material for: Beta-Defensin 2 and 3 Promote Bacterial Clearance of Pseudomonas aeruginosa by Inhibiting Macrophage Autophagy through Downregulation of Early Growth Response Gene-1 and c-FOS
Source: Front Immunol. 2018 Feb 13;9:211. doi: 10.3389/fimmu.2018.00211 (PMC5816924; doi:10.3389/fimmu.2018.00211)
Supplement: Supplementary file 1 [file Table_1.DOC]

Table 1. Nucleotide sequences of the specific primers used in PCR amplification.

| Gene*^a^* | Primer sequence (5’–3’) | Orientation *^b^* |
| --- | --- | --- |
| h-BD2 | TGTATCTCCTCTTCTCGTTCC  GGGCAAAAGACTGGATGAC | F  R |
| h-BD3 | TGTTTGCTTTGCTCTTCCTG  ACTTGCCGATCTGTTCCTC | F  R |
| m-BD2 | TCTCTGCTCTCTGCTGCTGATATGC  AGGACAAATGGCTCTGACACAGTACC | F  R |
| m-BD3 | TCTTTGCATTTCTCCTGGTGGTGC  AACTGCCAATCTGACGAGTGTTGC | F  R |
| h-EGR1 | CCTGACATCTCTCTGAACAACG  GGGAAAAGCGGCCAGTATAG | F  R |
| h-c-FOS | CCAGTGCCAACTTCATTCC  ATGGTCTTCACAACGCCAG | F  R |
| h-CR | AGCCCCTAGATGTACAGTGA  AGACCTGCCTTTTAATCGGA | F  R |
| h-FcγR | TCCATCCCACAAGCAAACCA  GCAATGACCACAGCCACAAT | F  R |
| h-MR | TGCTACTGAACCCCCACAA  AGAGGAACCCATTCGAAGACA | F  R |
| h-SR | CTGCTCCGAATCTGTGAAAT  GATGAGAACTGCAAACACGA | F  R |
| h-β-Actin | GCTCCTCCTGAGCGCAAG  CATCTGCTGGAAGGTGGACA | F  R |
| m-β-Actin | GATTACTGCTCTGGCTCCTAGC  GACTCATCGTACTCCTGCTTGC | F  R |
| h-EGR1- pSG5 | CCGGAATTCACACCAGCTCTCCAGCCTGCT  CCGCTCGAGCAACCTCCATCTGACCTAAGAGGAACG | F  R |
| h-c-FOS- pSG5 | CCGGAATTCATGATGTTCTCGGGCTTCAACGC  GAAGATCTTCACAGGGCCAGCAGCGTGGGTGA | F  R |

*^a^*h, human; m, mouse; *^b^*F, forward; R, reverse.
