# Supplementary material for: Beta-Defensin 2 and 3 Promote Bacterial Clearance of Pseudomonas aeruginosa by Inhibiting Macrophage Autophagy through Downregulation of Early Growth Response Gene-1 and c-FOS
Source: Front Immunol. 2018 Feb 13;9:211. doi: 10.3389/fimmu.2018.00211 (PMC5816924; doi:10.3389/fimmu.2018.00211)
Supplement: Supplementary file 2 [file Image_1.PDF]

Beta-defensin 2 and 3 promote bacterial clearance of *Pseudomonas aeruginosa* by inhibiting macrophage autophagy through down-regulation of EGR1 and c-FOS

Yongjian Wu [1, 2, ¶], Dandan Li [1, 3, ¶], Yi Wang [1, 3], Yuanqing Zhang [4], Wenting Qu[1, 3], Kang Chen[5], Ngiambudulu M. Francisco[1, 3], Lianqiang Feng[1, 3], Xi Huang [1, 3], MinhaoWu [1, 3,*]

1. Department of Immunology, Institute of Tuberculosis Control, Zhongshan School of Medicine, Sun Yat-sen University, Guangzhou 510080, China

2. Department of Gastroenterology, Guangzhou Women and Children's Medical Center, Guangzhou 510120, China

3. Key Laboratory of Tropical Diseases Control (Sun Yat-sen University), Ministry of Education, Guangzhou 510080, China

4. School of Pharmaceutical Sciences, Sun Yat-sen University, Guangzhou 510006, China

5. Division of Clinical Laboratory, Zhongshan Hospital of Sun Yat-sen University, Zhongshan 528403, China

[¶] The authors contributed equally to this article.

* Corresponding author

Minhao Wu, Ph.D.

Sun Yat-sen University Zhongshan School of Medicine,

74 Zhongshan 2nd Road, Guangzhou 510080, China

Phone: (86)20-87334049 e-mail: wuminhao@mail.sysu.edu.cn

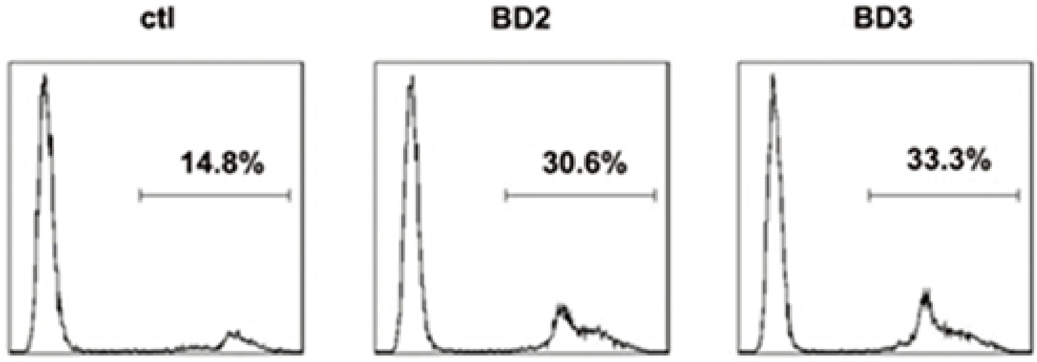

Supplementary Figure 1. BD2 and BD3 promoted macrophage-mediated phagocytosis of PA. THP-1 macrophages were treated with BD2 or BD3 for 6 h, and then infected with FTGB-labeled PA at MOI 25 for 1 h. Phagocytosis of PA was determined by flow cytometry.

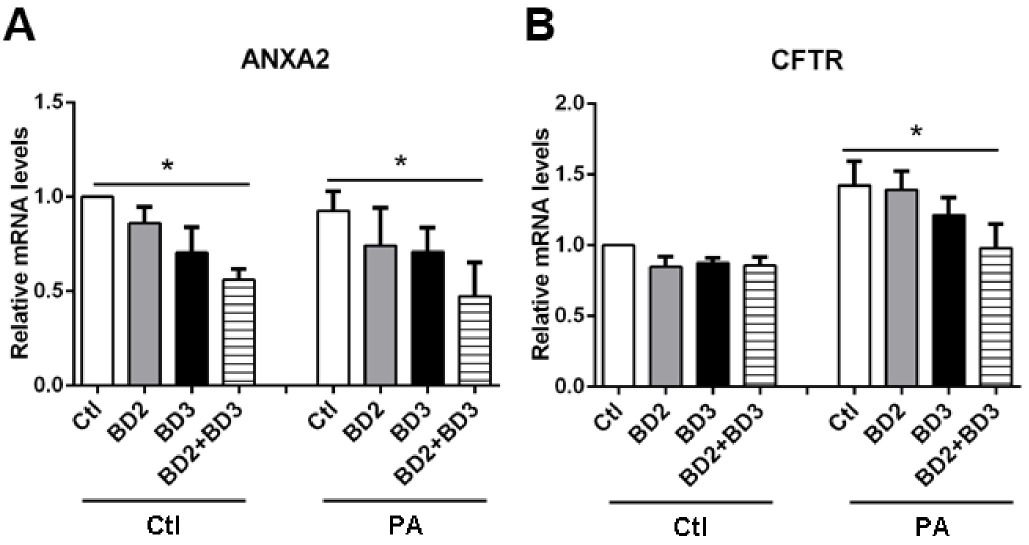

Supplementary Figure 2. **BD2 and BD3 had no significant effects on CFTR and ANXA2 expression**. THP-1 cells were treated with BD2 or BD3 or both peptides for 6 hours, followed by PA infection (MOI=1) for 6 hours. ANXA2 (A) and CFTR (B) mRNA levels were determined by real-time PCR. Data are shown as the mean ± SEM of three independent experiments.* $p<0.05$
